# Propionic acid produced by *Cutibacterium acnes* fermentation ameliorates ultraviolet B-induced melanin synthesis

**DOI:** 10.1038/s41598-021-91386-x

**Published:** 2021-06-07

**Authors:** Hsin-Jou Kao, Yan-Han Wang, Sunita Keshari, John Jackson Yang, Shinta Simbolon, Chun-Chuan Chen, Chun-Ming Huang

**Affiliations:** 1 Department of Biomedical Sciences and Engineering, National Central University, Zhongda Rd, No. 300, Zhongda Rd., Zhongli District, Taoyuan City, 32001 Taiwan, ROC; 2 Department of Dermatology, University of California, San Diego, CA USA; 3 Department of Life Sciences, National Central University, Zhongli District, Taoyuan City, Taiwan, ROC

**Keywords:** Biological techniques, Biotechnology, Microbiology

## Abstract

Ultraviolet irradiation induces melanin accumulation, which can be reduced by the use of chemical whitening products. However, the associated safety concerns of such products have prompted the search for natural and harmless alternatives. This study aimed to identify a natural acidic formulation to reduce skin pigmentation. The metabolite propionic acid (CH_3_CH_2_COOH, PA) was the most abundant fatty acid in the filtrate from Pluronic F68 (PF68) fermentation of *Cutibacterium acnes* (*C. acnes*) and reduced the DOPA-positive melanocytes by significantly inhibiting cellular tyrosinase activity via binding to the free fatty acid receptor 2 (FFAR2). Moreover, 4 mM PA treatment did not alter melanocyte proliferation, indicating that it is an effective solution for hyperpigmentation, causing no cellular damage. The reduced DOPA-positive melanocytes and tyrosinase activity were also observed in mice ear skin tissue injected with a mixture of *C. acnes* and PF68, supporting that the inhibition of melanogenesis is likely to be mediated through fermentation metabolites from *C. acnes* fermentation using PF68 as a carbon source. Additionally, PA did not affect the growth of its parent bacteria *C. acnes*, hence is a potent fermentation metabolite that does not disrupt the balance of the skin microbiome.

## Introduction

Skin acts as an interface between the human body and the external environment providing defense against pathogens, physical and ultraviolet damage^1^. If human skin is over exposed to ultraviolet radiation (UVR) that influences the function and survival of many cell types, inducing skin inflammation, which can lead to cancer^2,3^. UV-induced DNA damage activates cellular repair signals to produce melanin in melanocytes, resulting in skin pigmentation^4,5^. The healthy human skin surface is colonized by a diverse milieu of microorganisms, many of which are harmless as commensals or opportunistic pathogens^6,7^. Probiotics are common examples of bacteriotherapy with the potential for photoprotection by slowing the signs of aging skin and mitigating the effects of UV-induced skin inflammation^3,8,9^. For example, probiotic lactic acid bacteria notably prevents UV-induced inflammatory, allergic reactions, and immunosuppression in the skin^10^. Moreover, the ability of *Cutibacterium acnes* (*C. acnes*)*,* a predominant bacterium in the skin microbiome, to produce porphyrin in response to UVR makes it a major biomarker owing to its role in protecting and preventing disequilibrium^2,11,12^, hence it is of practical interest^13^. Studies revealed that galactomyces ferment filtrate (GFF) reduced melanin in human melanoma cells, thereby reducing skin pigmentation^14^. Free fatty acids (FFA) have remarkable regulatory effects on melanogenesis and suppressed tyrosinase activity in cultured B16F10 murine melanoma cells^15^. Most treatment options for hyperpigmentation block the conversion of tyrosine to melanin, acting as a tyrosinase inhibitor, a key regulatory enzyme required for melanin biosynthesis^16^. Recently, the extensive use of skin whitening agents, like phenolic and mercury compounds, in cosmetic formulations have raised serious safety concerns^17^. Moreover, FFAR2 (also known as GPR43) is present in a variety of tissues like bone marrow, spleen, and normal skin^18^ and is a promising drug targets for obesity, colitis, and some inflammatory responses^19^. FFAR2 is a receptor for short-chain fatty acids (SCFAs) with chain lengths less than six carbons such as acetate, butyrate, and propionate, the most potent agonist for FFAR2^20,21^. Previously, we demonstrated that in vivo knockdown of FFAR2 in mouse skin blocked butyric acid mediation of UV irritation^3^.

*C. acnes* is abundant on the human skin surface accounting for > 60% of the bacteria^2^. Moreover, propionic acid (PA), a fermentation metabolite from *C. acnes,* and its esterified derivatives, act as a potential antimicrobial agent against *Staphylococcus aureu*^11,22^ and poly(ethylene oxide) coatings are a promising method to avoid infections^23^. Skin commensal probiotic bacteria can inhibit the growth of USA300 through fermentation of poly(ethylene glycol) dimethacrylate as a selective fermentation initiator^24^. PF68, PEG-based polymer is a stable gel carrier for antimicrobial agents, increasing the surface solubility of the drug, and is usually used in the treatment of infected wounds without any discernible side effects^25^. In this study, we determined that PF68 as a polymer derived compound is a potentially safe and effective agent and the application of metabolites from the fermentation of PF68 by the skin commensal *C. acnes* could inhibit UV-induced hyperpigmentation or melanogenesis of skin.

## Methods

### Ethics statement

This study was carried out in strict with an approved Institutional Animal Care and Use Committee (IACUC) protocol at National Central University (NCU), Taiwan (NCU-106-016) and in compliance with the Arrive guidelines (https://arriveguidelines.org/). Institute Cancer Research (ICR) mice (8–9 weeks old females; National Laboratory Animal Center, Taipei, Taiwan) were sacrificed using CO_2_ in a closed box. All methods were performed in accordance with relevant guidelines and regulations.

### Bacterial culture

*C. acnes* (ATCC 6919) was cultured on Reinforced Clostridium Medium (RCM, Oxford, Hampshire, England) under anaerobic conditions using a Gas-Pak (BD, Sparks, MD, USA). Bacteria were cultured at 37 °C until the logarithmic growth phase. Bacterial pellets were harvested by centrifugation at 5,000 × g for 10 min, washed in phosphate-buffered saline (PBS), and then suspended in PBS or RCM for further experiments.

### Bacteria fermentation

*C. acnes* (10^5^ colony-forming unit (CFU)/mL) was incubated in 10 mL TSB in the presence or absence of 2% PF68 (BASF, NY, USA) under anaerobic conditions using Gas-Pak at 37 °C. PF68 alone in TSB was included as a control. The 0.002% (w/v) phenol red (Sigma) in TSB served as a fermentation indicator. A color change from red–orange to yellow indicated the occurrence of bacterial fermentation, which was detected by optical density at 560 nm (OD_560_).

### Gas chromatography‑mass spectrometry (GC–MS) analysis

*C. acnes* (10^5^ CFU/mL) was incubated in TSB (10 mL) in the presence of 2% PF68. After 1 day fermented media was centrifuged at 5000 g for 10 min and the supernatant was used for detection of SCFAs using previously published protocol^26^. The levels of acetic acid (AA), PA, butyric acid (BA), and iso-butyric acid (I-BA) in the fermentation media were quantified by a calibration curve made from six non-zero levels using the FFA Test Standard (Restek Corporation, Bellefonte, PA, USA) which is diluted to 500-, 1,000-, 2,000-, 5,000- and 10,000-fold.

### B16F10 melanoma cell culture

B16F10 melanoma cell line was kindly provided by Dr. Richard Gallo, University of California San Diego, USA, and Dr. Cheng Ching-Yi, Chang Gung University of Science and Technology, Taiwan. The cells were cultured in Dulbecco’s Modified Eagle’s Medium (DMEM, Life Technologies, Carlsbad, CA, USA) supplemented with 10% fetal bovine serum (FBS, Life Technologies) and 1% Penicillin–streptomycin (Life Technologies) at 37 °C and 5% CO_2_. The cells were grown until 70–80% confluence and then subcultured with Trypsin-Ethylenediaminetetraacetic acid (EDTA, Life Technologies).

### Reverse transcription-quantitative polymerase chain reaction (RT-qPCR)

RT-qPCR was employed to study the tyrosinase gene expression in B16F10 melanoma cells treated with 4 mM PA for 48 h. Total cellular RNA was extracted using a Quick-RNA MiniPrep Kit (Zymo research, CA, USA), followed by reverse transcription to cDNA using iScript cDNA synthesis kit (Bio-Rad, Hercules, CA, USA) and amplified by RT-qPCR in an ABI 7300 system (Applied Biosystems, Waltham, MA, USA). The comparative cycle threshold (ΔΔCT) was used to determine the quantification of gene expression. The gene-level of glyceraldehyde 3-phosphate dehydrogenase (GAPDH) was used for the normalization of tyrosine kinase gene. Primer for tyrosinase and GAPDH are 5′-TGACAAAGCCAAAACCCCCA-3′ (forward); 5′-TTGTTCAAAAATACTTCCAGTGTGT-3′ (reverse) and 5′-TGTGTCCGTCGRGGATCTGA-3′ (forward); 5′-GATGCCTGCTTCACCACCTT3′ (reverse).

### Cellular tyrosinase activity following FFAR2 gene knockdown

B16F10 melanoma cells were seeded at a density of 5 × 10^4^ cells/well in 24-well plates. Further, the cells were treated with FFAR2 selective antagonist GLPG0974 (0.1 µM, GLPG) and PA (4 mM) and incubated at 37 °C in 5% CO_2_ for 24 h. Cells were trypsinized and lysed with RIPA buffer (Thermo Fisher Scientific, NJ, USA). The lysed cells were frozen at -80 °C and thawed twice followed by centrifugation at 12,000 rpm for 10 min. The supernatant was mixed with 1 μg/mL of Levodopa (L-DOPA, Sigma) in 2:1 ratio in a 96-well plate, incubated at room temperature (RT) for 1 h and OD at 475 nm was detected^27^.

### BrdU labeling

B16F10 melanoma cells were grown on 8-well chamber slides (Thermo Fisher Scientific) in DMEM with 10% FBS, penicillin and streptomycin till 70% confluency achieved. Bromodeoxyuridine (10 μM, BrdU, ACROS, New Jersey, USA) was added to the culture media along with 4 mM PA. BrdU added with PBS in culture media was included as a control. After 24 h, cells were fixed with 4% perfluoroalkoxy (PFA, Sigma) and then permeabilized with 0.2% triton X-100 (Sigma) for 10 min. Next, cells were treated with 2 N HCl at 37 °C for 25 min, neutralized with HCl with 0.1 M Borate buffer (pH 8.5) for 10 min, and blocked with blocking buffer before incubation anti-BrdU antibody (Abcam, MA, USA) overnight and donkey anti-goat Alexa Fluor 568 IgG (H + L) (Life technologies) as second antibodies for 1 h at 4 °C. Nuclei were counterstained with 4,6-diamidino-2-phe-nylindole (DAPI, Sigma). Images were acquired with cellSens software (https://www.olympus-lifescience.com.cn/en/software/cellsens/; Version 1.2.1) connected to an Olympus BX63 microscope (Olympus, Tokyo, Japan).

### The mouse models created by UV light exposure

Mouse models have been instrumental in advancing the understanding of the many roles of UVR^28^. UVR increases DOPA-positive melanocytes in the skin, specifically at the site of exposure^29^. The protocol for the treatment of *C. acnes* injection to the mice ears has been described in our previous study^3^ (This reference does not talk about *C. acnes* injection). The ears of ICR mice were injected intradermally with *C. acnes* (10^7^ CFU) with and without 2% PF68 followed by ultraviolet B (UVB) exposure of 312 nm wavelength at a dose of 200 mJ/cm^2^ using an UV lamp (Model EB-280C, Spectronics Corp., Westbury, NY, USA) for 2 min every day for 3 days. Mouse ears injected with PBS or PF68 followed by UVB exposure were included as a control. In another group of experiment mice, ears were applied with 4 mM PA followed by UV exposure, with PBS as control.

### siRNA-Mediated gene silencing of FFAR2

In order to silence FFAR2 gene, we used the chemically-modified siRNA that targets FFAR2 receptor (FFAR2 siRNA), the siRNA negative control (NC siRNA) and the control without injection (C), which were obtained from GenePharma Co. (Shanghai, China). Their oligonucleotide sequences are siFFAR2: sense strand, 5′-CCGGUGCAGUACAAGUUAUTT-3′; anti-sense strand, 5′-AUAACUUGUACUGCACCGGTT-3′. SiControl: sense strand, 5′-UUCUCCGAACGUGUCACGUTT-3′; anti-sense strand, 5′-ACGUGACACGUUCGGAGAATT-3′. These chemically-modified siRNAs were delivered every day for 3 days by intradermal injection in the ears of mice using a microneedle (2 mg/kg of mice weight). From day two, apply with 2% PA and UV exposure for 3 days. The pretreatment for injecting siRNA to the mouse as described in reference^30^. Then, mice ears were excised and stained on the fourth day.

### Western blotting

Mice ears were injected with chemically-modified FFAR2 and control siRNAs followed by application with 2% PA and UVB exposure for 3 days. On third day mice ears were cut, homogenized and then lysed with RIPA buffer (Thermo Fisher Scientific). Cell lysates (30 µg) were subjected to 10% SDS-PAGE gel, which were then transferred to a poly(vinylidene fluoride) (PVDF) membrane (Sigma) and blocked with 5% (w/v) nonfat milk before incubation overnight with primary antibodies to FFAR2 Rabbit PolyAb (Proteintech, Rosemont, IL, USA) at 4 °C or β-actin (1:1,000; Cusabio Technology, Houston, TX, USA). This was followed by treatment with horseradish peroxidase-conjugated goat anti-rabbit secondary antibody (1:5000) (Thermo Fisher Scientific) for 1 h. Protein bands were detected with a chemiluminescent detection reagent (Thermo Fisher Scientific) and Omega Lum C Imaging System (Gel Co., San Francisco, CA, USA). Protein bands were conducted using ImageJ software (https://imagej.nih.gov/ij/; Version 1.53e).

### Melanocyte counting

The cartilages from mouse were removed manually and the skin tissues were soaked in ammonium thiocyanate (Sigma) solution at 37 °C for 20 min. The epidermal and basal layers were exfoliated from the rest of the skin tissue, and melanocytes were stained by immersing in 0.1 M PBS (pH 7.2) containing 0.14% L-DOPA at RT for 3 h and the melanocyte count in skin tissues was determined microscopically according to a previously published protocol^29^.

### Statistical analyses

Data analysis was performed by unpaired t-test using Prism software (https://www.graphpad.com/; Version 5.01, GraphPad Software, La Jolla, CA, USA). The levels of statistical significance were indicated as the following: *P < 0.05, **P < 0.01, ***P < 0.001 and ns = non-significant. The mean ± standard deviation (SD) for at least three independent experiments except two independent western blotting analysis for Fig. 4C was displayed. Animal experiments were performed with at least three animals per treatment group.

## Results

### *C. acnes* induces fermentation of PF68

**Figure 1:**
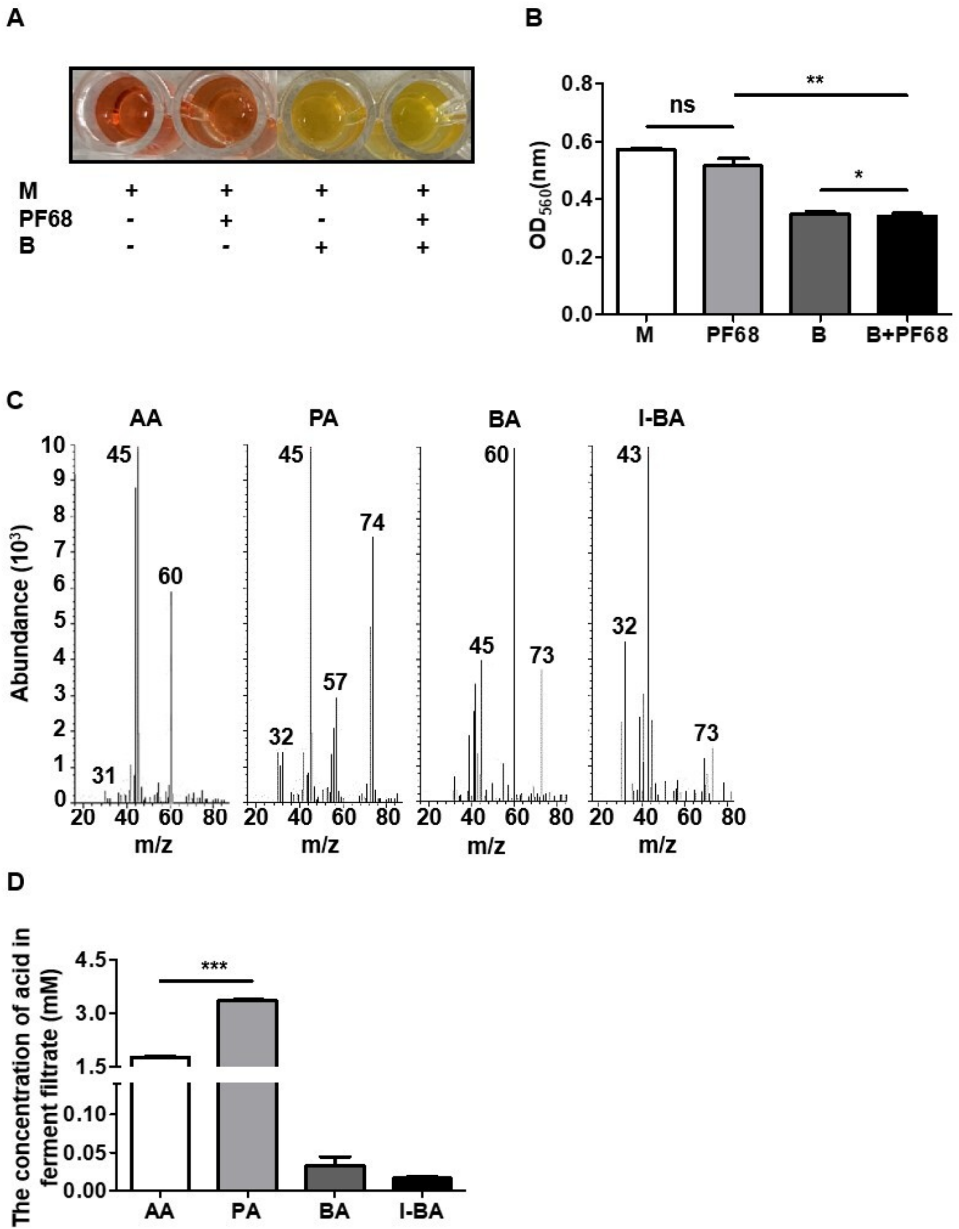
The bacterial fermentation with polymer was accompanied by the decrease in intracellular pH. (**A**)The color change of phenol red from red to yellow when *C. acnes* was incubated with/without PF68 in TSB. (**B**) The prevalence of fermentation was quantified by measuring the optical density of phenol red at OD_560._ The pH of media with PF68 and *C. acnes* was significantly lower than the other groups. (**C**) The ion chromatogram and mass spectrum showing the SCFAs present in the *C. acnes* ferment cultured with PF68. (**D**) Quantification of SCFAs in the *C. acnes* ferment filtrate. A high amount of PA was produced by PF68 fermentation of *C. acnes*. Medium (M), *C. acnes* (B), acetic acid (AA), propionic acid (PA), butyric acid (BA), and iso-butyric acid (I-BA). Data are the mean ± SD from three separate experiments. *P < 0.05, **P < 0.01, ***P < 0.001 and ns = non-significant (two-tailed t-test).

Previous studies have demonstrated the fermentation of skin probiotics for induction of SCFA production in the presence of compounds as a carbon source^26^. To investigate if *C. acnes* could ferment PF68, *C. acnes* was incubated with PF68 in TSB media for 1 day with phenol red as an indicator to the monitor the bacterial fermentation. In TSB media incubated with bacteria alone, the phenol red changed from red to orange due to bacterial replication, whereas in the TSB media containing bacteria and PF68, the phenol red color changed to pale yellow with a decrease in pH indicating the use of PF68 as a carbon source for fermentation (Fig. 1A). Furthermore, the OD_560_ of phenol red showed a significant decrease in pH value in the TSB media containing bacteria and PF68 (Fig. 1B) compared to bacteria or PF68 alone. The SCFAs contained in the *C. acnes* ferment cultured with PF68 were identified by GC–MS analysis (Fig. 1C) and found to be AA, PA, BA, and I-BA acid, with PA^11^ having the highest concentration (Fig. 1D).

### In vitro effect of PA on melanin production via PA-FFAR2

**Figure 2:**
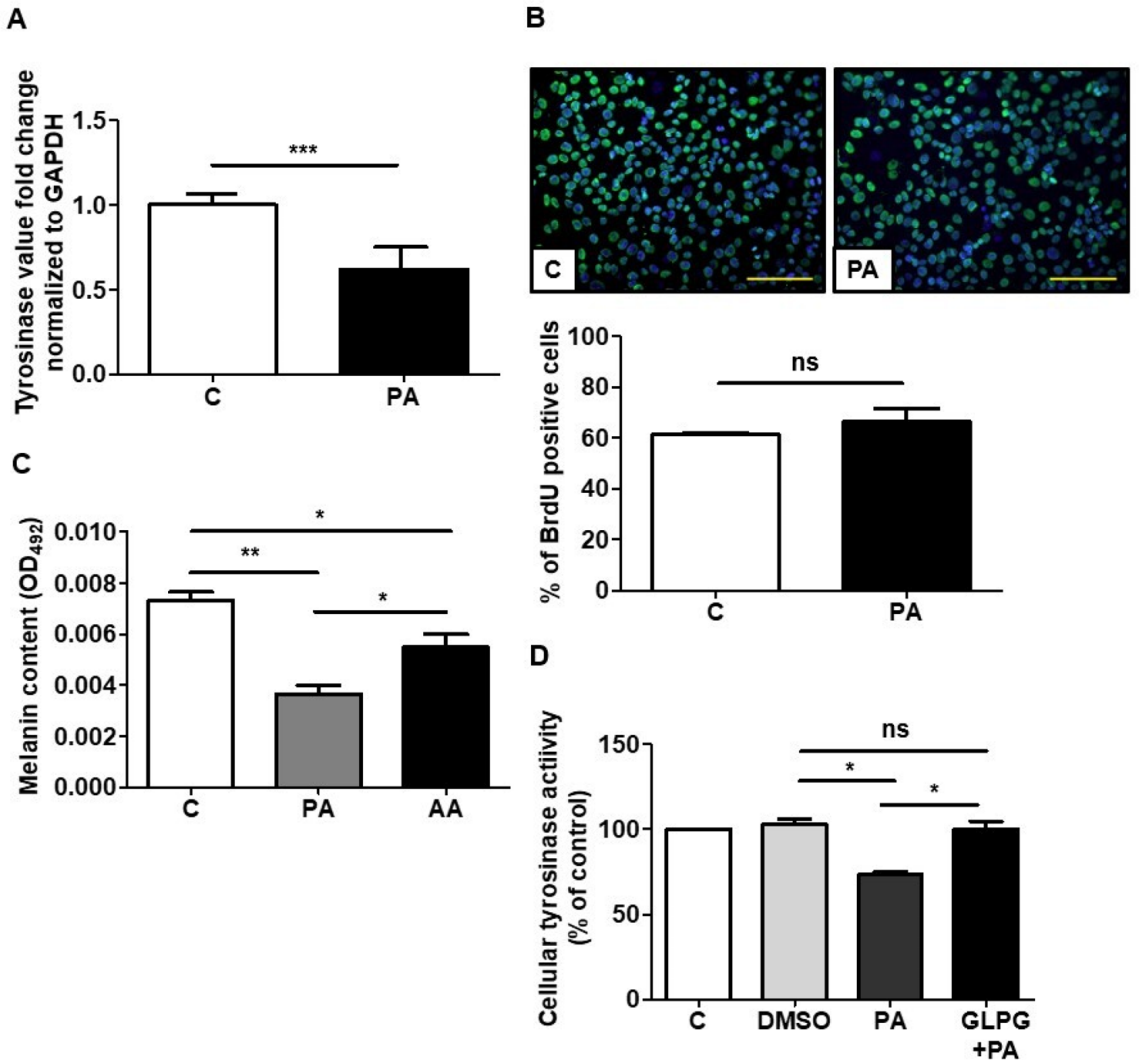
The effects of tyrosinase gene expression and cell proliferation in melanoma cells after PA treatment. (**A**) Tyrosinase gene expression decreased in PA treated B16F10 melanoma cells quantified by RT-qPCR. (**B**) The effects of PA on B16F10 melanoma cells evaluated by BrdU labeling. PA treatment did not alter the cell proliferation. (**C**) Effects of PA and AA in melanocytes. The relatively higher efficacy of PA in attenuating melanin content. (**D**) Blocking the signaling of FFar2 using GLPG prevents the PA mediated decreased cellular tyrosinase activity in B16F10 cells. Control (C), propionic acid (PA), acetic acid (AA), dimethyl sulfoxide (DMSO), and GLPG0974 (GLPG). Data are the mean ± SD from three separate experiments. *P < 0.05, **P < 0.01 and ns = non-significant (two-tailed t-test). Scale bar = 120 µm.

Fatty acids modulate the degradation of tyrosinase, a crucial enzyme involved in melanin biosynthesis in melanocytes and melanoma cells^31^. Therefore, to detect the in vitro effect of PA on melanin synthesis, B16F10 melanoma cells were treated with 4 mM PA for 48 h, with the decreased expression of the tyrosinase in PA treated melanoma cells compared to the control (Fig. 2A). The immunostaining of PA treated melanoma cells after BrdU labeling showed that PA did not alter the proliferation of melanoma cells (Fig. 2B). Furthermore, the effect of PA and AA, two highly expressed SCFAs in the fermentation filtrate (Fig. 1D)^32^, on melanin production was assessed in melanoma cells, with PA causing a > twofold decrease in melanin production compared to AA (Fig. 2C). SCFAs, like PA and AA, regulate their functions via interaction with FFAR2 and FFAR3, with PA being among the most potent SCFA for both these receptors^33^. Considering PA regulation via its interaction with FFAR2, blocking the signaling of this receptor using a selective inhibitor could be a useful approach to evaluate the role of PA in the regulation of melanogenesis. The cellular tyrosinase activity was unchanged by blocking FFAR2 using the FFAR2 selective antagonist GLPG before treatment with PA and decreased without GLPG in contrast to the control group (Fig. 2D). These results demonstrated the effective role of PA-FFAR2 in the attenuation of the melanin content by inhibiting tyrosinase kinase activity in melanoma cells with unaltered cell proliferation.

### The mixture of *C. acnes* and PF68 inhibited UVB-induced functioning melanocyte in mouse ear

**Figure 3:**
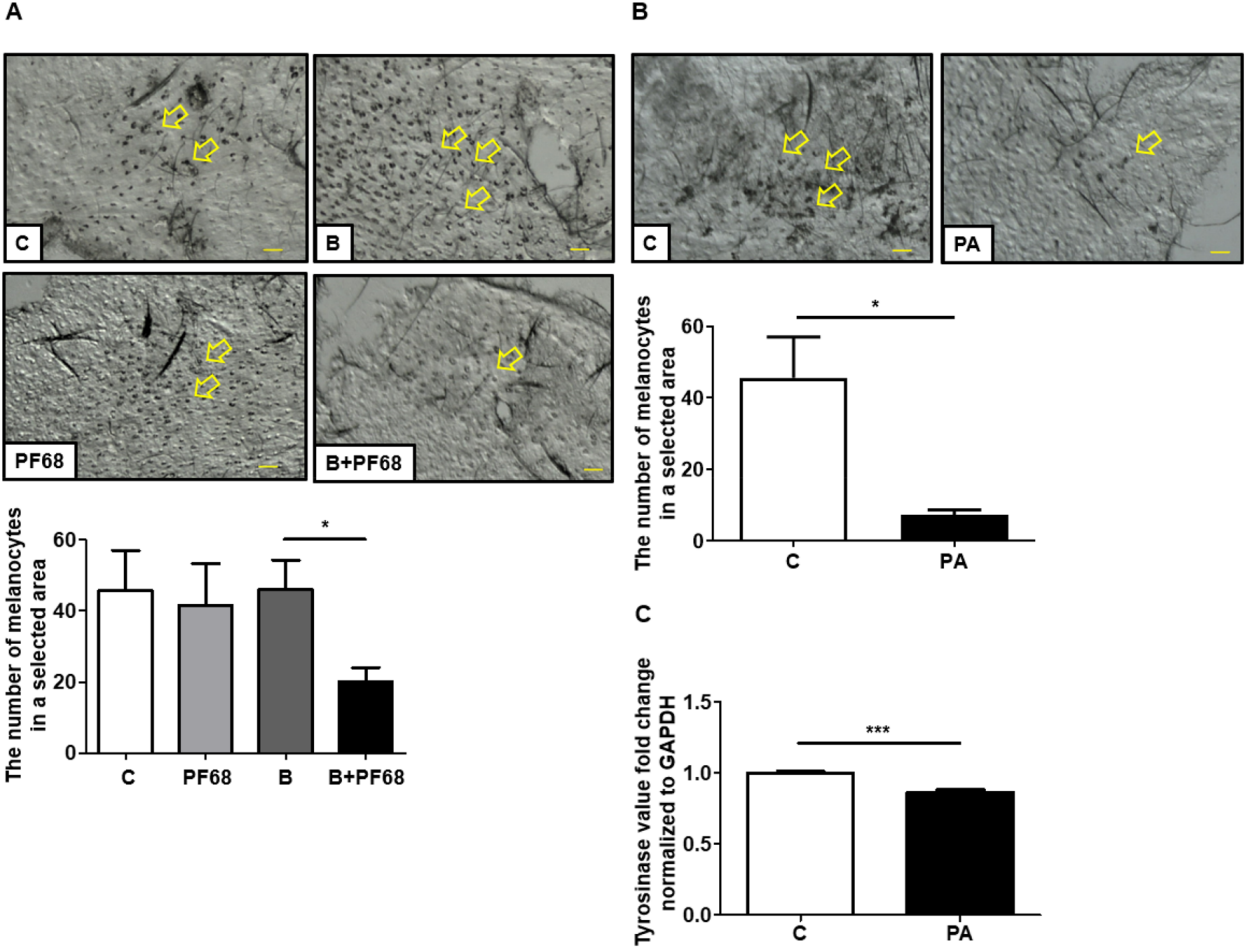
Induction of melanocytes by UVB and inhibition by different treatments. (**A**) The ears of mice were applied PF68 + *C. acnes*. The same amount of PBS, PF68 only, and *C. acnes* only were applied as control. Specimens were examined microscopically for the effects of PF68 + *C. acnes* treatment on the DOPA-positive melanocytes proliferation in the epidermis. (**B**) The ears of mice were applied PA. The same amount of PBS was applied as control. Specimens were examined microscopically for the effects of PA treatment on the DOPA-positive melanocytes proliferation in the epidermis. (**C**) Tyrosinase gene expression after PA treatment in vivo was quantified by RT-qPCR. Control (C), *C. acnes* (B), propionic acid (PA). The arrowhead in A and B shows the staining of DOPA-positive melanocytes that was counted in the same millimeter squared. Data are the mean ± SD from three separate experiments was replayed. *P < 0.05, ***P < 0.001 (two-tailed t-test). Scale bar = 60 μm.

The topical application of fermentation extracts or fatty acids has been shown to decrease UV-induced skin hyperpigmentation by regulating tyrosinase degradation^34,35^. Previous studies demonstrated that melanogenesis after UV exposure is due to the activation in functioning melanocytes in the epidermis or dermis^36^. Having established that PA as a major SCFA from *C. acnes* fermentation of PF68 could effectively reduce melanin production in vitro, we then examined the effect of *C. acnes* plus PF68 on it in vivo. The ears of ICR mice were exposed to UVB every other day for 3 days concurrent with the injection of *C. acnes* and PF68. Injection with PBS or *C. acnes* or PF68 alone were included as controls. The epidermal and basal layers were exfoliated from the skin tissue in the mouse ear and the melanocytes were stained with L-DOPA. There was a significant increase of the melanocytes number after UVB exposure, with no change after injection with *C. acnes* or PF68 alone. However, a significant reduction in number of DOPA-positive melanocytes was detected in the UVB exposure groups after treatment with a mixture of *C. acnes* and PF68 (Fig. 3A).

### Amelioration of UVB-induced functioning melanocyte in mouse ear by PA, a *C. acnes* fermentation metabolite

The effect of the direct application of PA on UV-induced melanogenesis was assessed. UV-induced hyperpigmentation and tyrosinase expression in mouse ear skin in the control group was significantly lowered after the topical application of PA for 3 days (Fig. 3B). Thus, PA, a metabolite from PF68 fermentation of *C. acnes* inhibits functioning melanocytes production with melanin synthesis by UVB-induced. Tyrosinase expression was noticeably decreased in PA treated cells compared to the control (Fig. 3C).

### PA inhibits UVB-induced functioning melanocytes via FFAR2 in vivo

**Figure 4:**
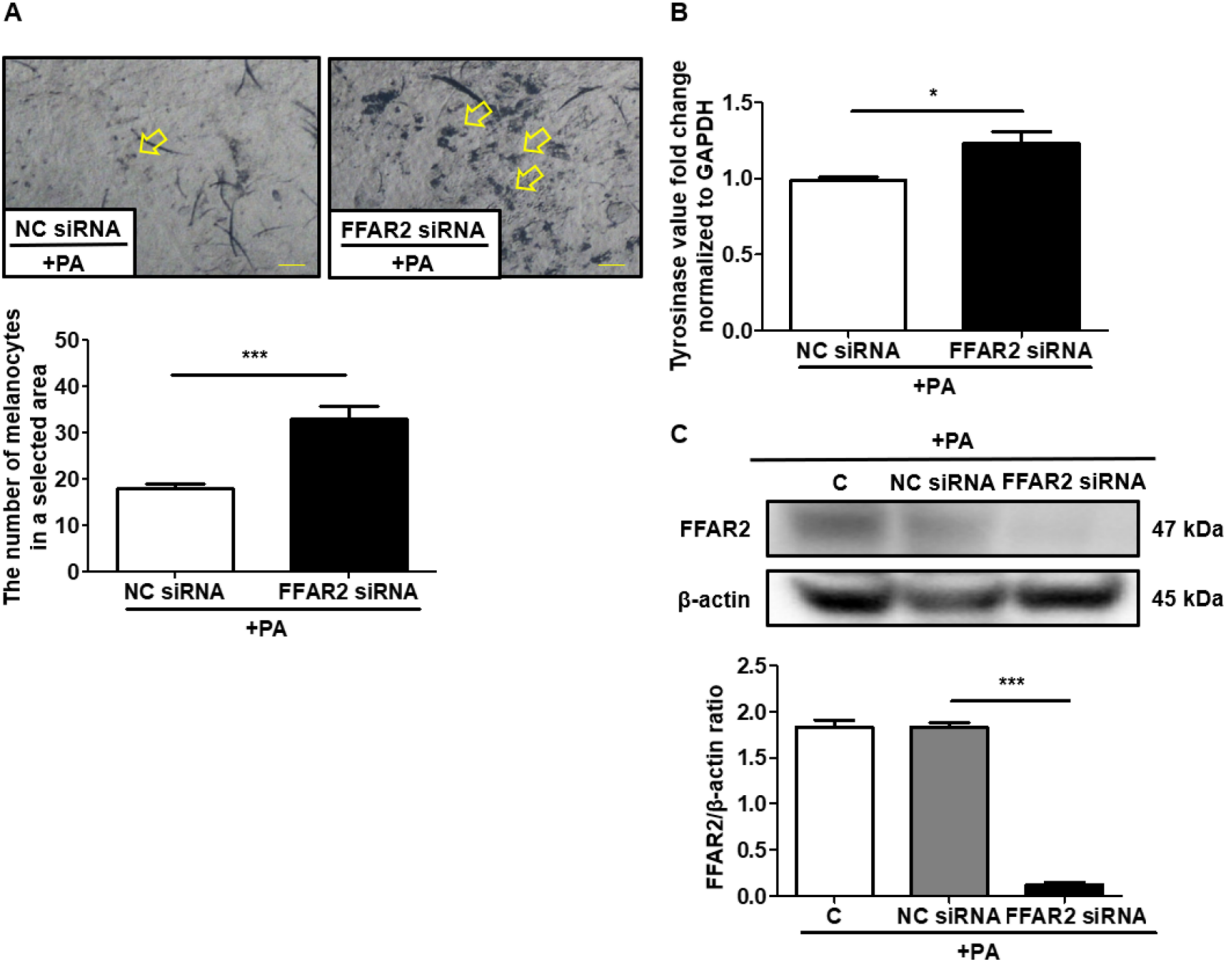
The siRNA knockdown efficiency of the FFAR2 gene, along with the topical application of PA and UV exposure to ICR mice ears. (**A**) Specimens were examined microscopically for the effects of PA treatment after FFAR2 gene knockdown on the DOPA-positive melanocytes proliferation in the epidermis. (**B**) Tyrosinase gene expression in vivo quantified by RT-qPCR. (**C**) Western blotting to confirm FFAR2 gene knockdown and the expression of FFAR2 and β-actin in mice ears. Full-length gels images are available in Supplemental Information. Control (C), propionic acid (PA), negative control (NC). The arrowhead in A and B shows the staining of DOPA-positive melanocytes that was counted in the same millimeter squared. Data A and B are the mean ± SD from three separate experiments except two independent western blotting analysis for C was replayed. *P < 0.05, ***P < 0.001 (two-tailed t-test). Scale bar = 60 μm.

To determine if the reduction in exogenous melanogenesis occurred via PA interaction with its cognate receptor, FFAR2 was knocked down in the skin melanocytes of the mouse ear, followed by UVB exposure and PA treatment. UVB-induced an increase in the proliferation of melanocytes and tyrosinase activity was significantly reduced in the mouse ear epidermis injected with NC siRNA after application of PA (Fig. 4A,B). However, the number of melanocytes and tyrosinase activity remains unchanged even after PA application in UVB irradiated mouse ear epidermis knocked down with FFAR2. We confirmed the FFAR2 gene knockdown by measuring the protein expression level of FFAR2 by Western blot analysis (Fig. 4C). Thus, PA interferes with melanogenesis by suppressing the activity of tyrosinase, in which PA-FFAR2 plays a potential role in melanin production.

## Discussion

Melanin, a critical factor of skin defense against UV-irradiation, is synthesized in the melanosomes of melanocytes, transferred to neighboring keratinocytes via the dendritic tips, and eventually distributed throughout the skin epidermis^37^. Bacteria fermentation metabolites are increasingly used in skin therapy due to their beneficial effect on UV-induced skin inflammation and infectious disorders^3^. In this study, we demonstrated that PA, the major metabolite from PF68 fermentation of *C. acnes,* reduced UVB-induced functioning melanocyte levels by inhibiting tyrosinase activity in vitro and in vivo via FFAR2.

Previous studies demonstrated the inhibition of melanogenesis through tyrosinase inhibition by fermentation metabolites from probiotic LA and *Lactobacillus paracasei* bacteria^24,38,39^. Also, the high molecular weight PEG-based polymer (~ 20,000) was completely degraded through fermentation by gram-negative bacteria producing acetate and propionate^40^. In this study, PA (~ 4 mM) was the most abundant SCFA in the filtrate from PF68 fermentation of *C. acnes* and reduced the melanin content in melanocytes more effectively than AA. Furthermore, treatment with 4 mM PA significantly inhibited cellular tyrosinase activity in melanocytes proving that inhibition of melanogenesis by PA occurred via the reduction of tyrosinase gene expression. People have the same number of melanocytes that is not the reason to affect skin pigmentation but the activation in functioning melanocytes by UV-irradiation^36,41^. Here, 4 mM PA treatment did not alter melanocyte proliferation, indicating that it is an effective treatment option, causing no cellular damage. The reduced melanocytes number and tyrosinase activity were also observed in mice ear skin tissue injected with a mixture of *C. acnes* and PF68, demonstrating that the inhibition of melanogenesis is likely to be mediated through fermentation metabolites from *C. acnes* fermentation using PF68 as a carbon source. Additionally, our study validated PA as an excellent antimicrobial agent, with no alteration in the growth of its parent bacteria *C. acnes,* showing PA as a potent metabolite that does not disrupt the balance of the skin microbiome^11^.

Melanin production is initiated and regulated by several signaling systems, whereby tyrosinase catalyzes the conversion of tyrosine to DOPA, and into dopaquinone, leading to the formation of melanocytic pigments^42,43^. In most cases, skin lightening reagents act at various levels of melanin production via FFAR2 to affect tyrosinase activity or directly targeting tyrosinase to inhibit melanogenesis in the skin^44–46^. SCFAs such as PA and AA exert their effects through binding to their selective cognate receptors like FFAR2 or FFAR3, with PA being among the most potent SCFA for both receptors^47^. GLPG, a selective FFAR2 antagonist, and siRNA-mediated gene silencing of FFAR2 to support the blocking of FFAR2^3,48^. In the current study, the increased melanocytes number and tyrosinase gene expression in mice ear skin tissue did not changed in response to UVB radiation even after PA application to FFAR2 knockdown in mice (Fig. 4A). Furthermore, tyrosinase gene expression was decreased after PA treatment in vitro, however, blocking the FFAR2 gene with GLPG, a selective FFAR2 antagonist, and ameliorated the effect of PA to attenuate tyrosinase gene expression. After blocking FFAR2 as loss of FFAR2 results in enhanced expression of tyrosinase activity after PA treatment. Although both PA and AA, the fermentation metabolites from *C. acnes*, have a similar affinity for FFAR2 binding^20^, the relatively lower efficacy of AA in attenuating tyrosinase activity in melanocytes validates the therapeutic potential of PA as a postbiotic against melanogenesis.

The photo damaging effects caused by UVR on the skin have attracted extensive attention. Sun screening and anti-hyperpigmentation products are widely used but their safety, skin penetration, and therapeutic efficacy are still in question^49^. Although avobenzone is a common component in sunscreens owing to its high efficacy against UV, it is photo unstable and therefore not safe, and has been detected in blood after chronic applications^50,51^. Development of effective skin whitening through blocking tyrosinase expression by fermentation metabolites from probiotic bacteria or polymers as a carbon source is an effective and natural way to suppress melanogenesis^38,39^. As the use of live probiotics for cosmetics is strictly regulated, the promotion of their beneficial effects through fermentated metabolites from live probiotic bacteria has become a feasible application. Besides, PF68 as a prebiotic can boost the production of SCFAs from *C. acnes* fermentation and has been used to enhance the biological stability and assist various therapeutic agents such as 6-mercaptopurine loaded microspheres in their interaction with the human body^52^. Furthermore, PF68 can be incorporated into cell membranes and translocate into cells and has been used as a stable gel carrier for antimicrobial agents, increasing the surface solubility of the drug in skin treatment^12,23^. Hence, PF68 is considered a safe and effective options for the development of pharmaceuticals and cosmetics. In addition to the function of PF68 as a fermentation initiator to augment the fermentation activity of *C. acnes*, it also has the potential to be an adjuvant to reduce the effective doses of medicine reagents and improve the solubility of poor-water soluble drugs.

Overall, these results demonstrate that the PA-FFAR2 interaction may be a central mechanism in the effect of probiotics or postbiotics on hyperpigmentation caused by UVR. PA treatment did not affect melanocyte proliferation. Compared with chemical therapies, the metabolites of probiotics are milder and natural^53^. Although the exact mechanism by which the metabolites of PF68 fermentation of *C. acnes* inhibit melanogenesis is unclear, the evidence from the present study suggests the involvement of the SCFAs-FFAR2-tyrosinase pathway. These results are beneficial for the future clinical treatment of pigmentation disorders and to develop cosmetics that increases the range of applications of whitening products. Lastly, the diversity of probiotics offers personalized treatments for hyperpigmentation and the development of dedicated products with higher performance.

## Supplementary Information

Supplementary Information.
