# Supplementary material for: Propionic acid produced by Cutibacterium acnes fermentation ameliorates ultraviolet B-induced melanin synthesis
Source: Sci Rep. 2021 Jun 7;11:11980. doi: 10.1038/s41598-021-91386-x (PMC8184931; doi:10.1038/s41598-021-91386-x)
Supplement: Supplementary file 1 — Supplementary Information. [file 41598_2021_91386_MOESM1_ESM.docx]

**Supplemental Information**

Propionic acid produced by *Cutibacterium acnes* fermentation ameliorates ultraviolet B-induced melanin synthesis

Hsin-Jou Kao^1^, Yan-Han Wang^2^, Sunita Keshari^3^, John Jackson Yang^3^, Shinta Simbolon^1,^ Chun-Chuan Chen^1^, and Chun-Ming Huang^1*^

^1^ Department of Biomedical Sciences and Engineering, National Central University, Zhongli District, Taoyuan City, Taiwan (R.O.C.)

^2^ Department of Dermatology, University of California, San Diego, CA, USA

^3^ Department of Life Sciences, National Central University, National Central University, Zhongli District, Taoyuan City, Taiwan (R.O.C.)

***** Correspondence: chunmingsd@gmail.com; Tel.: +886-3-422-7151#36104; National Central University, No. 300, Zhongda Rd., Zhongli District, Taoyuan 32001, Taiwan (R.O.C.)


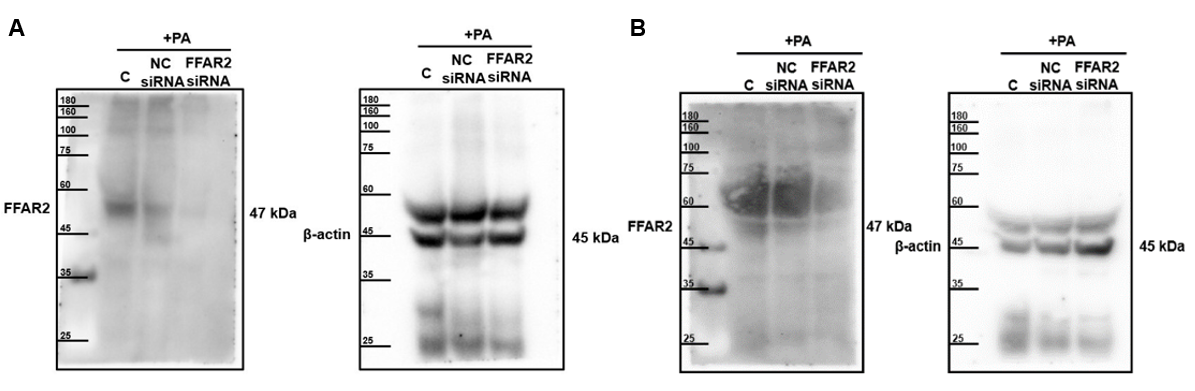


**Figure S1.** (A)The uncropped western blot images of Fig. 4C. (B) two separate western blotting analysis was displayed. The lanes from top to bottom of each blot represent the size of protein ladder (kDa); The protein band levels of FFAR2 & β-actin in the mice ear treated with and without FFAR2 siRNA
